# Glial cells in familial amyloidotic polyneuropathy

**DOI:** 10.1186/s40478-014-0177-8

**Published:** 2014-12-18

**Authors:** Nádia Pereira Gonçalves, Susete Costelha, Maria João Saraiva

**Affiliations:** Molecular Neurobiology, Instituto de Biologia Molecular e Celular – IBMC, Rua do Campo Alegre 823, Porto, 4150-180 Portugal; Instituto de Ciências Biomédicas de Abel Salazar (ICBAS), Universidade do Porto, Porto, 4050-313 Portugal

**Keywords:** Transthyretin, Internalization, Glial cells, Familial amyloidotic polyneuropathy, Peripheral nervous system, Myenteric plexus

## Abstract

**Introduction:**

Transthyretin V30M mutation is the most common variant leading to Familial Amyloidotic Polyneuropathy. In this genetic disorder, Transthyretin accumulates preferentially in the extracellular matrix of peripheral and autonomic nervous systems leading to cell death and dysfunction. Thus, knowledge regarding important biological systems for Transthyretin clearance might unravel novel insights into Familial Amyloidotic Polyneuropathy pathophysiology. Herein, our aim was to evaluate the ability of glial cells from peripheral and autonomic nervous systems in Transthyretin uptake and degradation. We assessed the role of glial cells in Familial Amyloidotic Polyneuropathy pathogenesis with real-time polymerase chain reaction, immunohistochemistry, interference RNA and confocal microscopy.

**Results:**

Histological examination revealed that Schwann cells and satellite cells, from an Familial Amyloidotic Polyneuropathy mouse model, internalize and degrade non-fibrillar Transthyretin. Immunohistochemical studies of human nerve biopsies from V30M patients and disease controls showed intracellular Transthyretin immunoreactivity in Schwann cells, corroborating animal data. Additionally, we found Transthyretin expression in colon of this Familial Amyloidotic Polyneuropathy mouse model, probably being synthesized by satellite cells of the myenteric plexus.

**Conclusions:**

Glial cells from the peripheral and autonomic nervous systems are able to internalize Transthyretin. Overall, these findings bring to light the closest relationship between Transthyretin burden and clearance from the nervous system extracellular milieu.

## Introduction

Familial Amyloidotic Polyneuropathy (FAP) is a rare but fulminant and life-threatening neurodegenerative disorder. Approximately ten thousand patients are affected worldwide with endemic foci in Portugal, Japan and Sweden [1]. Major neuropathological and neurochemical hallmarks of this autosomal dominant hereditary disease included extracellular accumulation of mutated transthyretin (TTR) aggregates and amyloid fibers, particularly in autonomic and peripheral nervous systems (ANS and PNS, respectively), leading to sensorimotor, motor and autonomic neuropathy [2].

Transthyretin is a tetramer of identical subunits of 127 amino acid residues each [3]. It is primarily synthesized by the liver and the choroid plexus of the brain [4,5] and functions as a protein carrier for thyroxin and retinol [6,7]. More than 100 single point TTR mutations have been discovered, being the exchange of a methionine for a valine at position 30 (V30M) the most common in FAP [8]. TTR is mainly produced by the liver as a monomer that assembles into a tetramer and is efficiently secreted. This process occurs in most FAP associated mutations, including carriers of the V30M mutation [9]. Particular high amyloidogenic mutations, such as the L12P associated with leptomeningeal amyloidosis, form intracellular aggregates that are transported into liver lysosomes [10] and thus these mutants are poorly secreted. Contrarily to other TTR amyloidoses, L12P cases present liver TTR deposition [11].

The original amyloid cascade hypothesis proposed that circulating TTR dissociation into non-native monomers is a determinating step for misfolding. Thus, monomers with low conformational stability self-assemble forming non-fibrillar oligomers, protofibrils and mature amyloid fibers [12,13] that accumulate in the extracellular matrix of the gastrointestinal tract, skin, heart, kidney and PNS [14].

A particular feature of FAP is organ and tissue tropism for TTR deposition. The mechanistic and functional principles that underlie this fact are not fully understood. Hence, converging evidence revealed the importance of a dynamic balance between formation and clearance of extracellular deposited TTR [15,16]. Cellular uptake of soluble TTR by hepatocytes, mouse embryonic fibroblasts, yolk sac cells and sensory neurons, was previously demonstrated *in vitro* [17-19]. More recently, intracellular material has been observed in fibroblasts and macrophages, through analysis of skin biopsies from FAP patients and TTR transgenic mice [20].

Since important target tissues of TTR load belong to the ANS and PNS, the aim of the present study was to investigate TTR localization in tissues and cells of these systems. To perform this work we take advantage of human nerve biopsies and tissues from a well-established FAP mouse model, carrying the human *TTR V30M* gene.

We analyzed *TTR* expression in the peripheral nerve of the FAP mouse model using real-time polymerase chain reaction (qPCR) analysis and determined the subcellular localization of TTR in satellite cells from mice dorsal root ganglia (DRG) and Schwann cells (SC). SC from patients and control subjects were also evaluated, through confocal double immunofluorescence. An interference RNA (RNAi) approach was used to study TTR expression/internalization by satellite cells from the myenteric plexus.

## Materials and methods

### Ethics statement

All mouse protocols followed the European Union Directive (2010/63/EU) and were previously approved by the Institutional and National General Veterinarian Board ethical committees. Human tissue biopsy was performed after informed consent and approval from the Ethics Committee of the Hospital Geral de Santo António (Porto, Portugal), following the declaration of Helsinki.

### Human samples

Archival sural nerve biopsy samples obtained from FAP V30M patients (*n* = 4) and normal disease control subjects (*n* = 4) were previously characterized, after informed consent, for TTR and amyloid deposition, with immunohistochemistry and Congo red staining, respectively. Samples were kindly provided by the Hospital Geral de Santo António (Porto, Portugal). FAP samples analyzed in this study presented TTR amyloid deposition. Disease control patients were near-relatives of FAP patients who ultimately turned out not to have mutations in TTR. This material was obtained as part of the clinical diagnosis and evaluation of polyneuropathy, before the current use of less invasive methods, as previously described [21].

### Animals

Six months transgenic mice for human *TTR V30M*, in the 129/Sv and endogenous *Ttr* null background, heterozygous for the heat shock factor-1 (*Hsf-1*), here designated as Hsf/V30M [22], were used for the experiments. Although not presenting amyloid fibers in PNS or ANS, non-fibrillar TTR material is widespread in the extracellular milieu of these systems at 6 months of age. Additionally, 6 months-old Ttr wild-type (WT) and TTR knockout (KO) mice [23], in a 129/Sv background were used as controls. Animals were housed in a controlled-temperature room, maintained under a 12 h light/dark cycle, with water and food *ad libitum* and euthanized with a lethal injection of a premixed solution containing ketamine (75 mg/kg) and medetomidine (1 mg/kg).

### Liver TTR silencing *in vivo* with RNAi

For *TTR*-silencing studies, TTR or control siRNA (vehicle) were formulated into a lipid nanoparticle delivery system [24]. Five months-old Hsf/V30M mice were injected in the tail vein with human TTR siRNA (*n* = 6), at a concentration of 1 mg/kg. Untreated age-matched controls received vehicle intravenously (*n* = 6). One injection per week was performed during 4 weeks and animals were sacrificed 48 h after the last injection. Liver and colon were divided and collected to 10% formalin and frozen at −80°C.

### Messenger RNA (mRNA) isolation, complementary DNA (cDNA) synthesis and real-time quantitative polymerase chain reaction (qPCR)

Liver and colon mRNA (*n* = 6 per group Hsf/V30M; *n* = 5 WT) was isolated using phenol extraction (Invitrogen, Carlsbad, CA, USA). Sciatic nerve from Hsf/V30M mice was dissected free from surrounding tissue (*n* = 5) and mRNA extraction performed using RNeasy Mini columns (Qiagen, Gaithersburg, MD, USA). cDNA was synthesized with the SuperScript double-stranded cDNA Kit (Invitrogen). The quality of extracted RNA was assessed with Experion RNA StdSens Analysis Kit (Bio-Rad, Hercules, CA, USA); qPCR was performed in duplicates using iQ Syber Green Super Mix (Bio-Rad) and reactions were run on an Bio-Rad iQ5 software.

Primer sequences were designed using Beacon Designer 8™ (Premier Biosoft, Palo Alto, CA, USA) for *TTR* (Forward: 5’-ATTCTTGGCAGGATGGCTTC-3’, Reverse: 5’-CAGAGGACACTTGGATTCACC-3’); *Ttr* (Forward: 5’-AGCCCTTTGCCTCTGGGAAGAC-3’, Reverse: 5’-TGCGATGGTGTAGTGGCGATGG-3’); glyceraldehyde 3-phosphate dehydrogenase *(Gapdh)* (Forward: 5’-GCCTTCCGTGTTCCTACC-3’, Reverse: 5’-AGAGTGGGAGTTGCTGTTG-3’) and *18S* (Forward: 5’-AAATCAGTTATGGTTCCTTTGGTC-3’, Reverse: 5’-GCTCTAGAATTACCACAGTTATCCAA-3’). Differential expression was determined by the 2^-^∆∆CT^ method.

### Immunohistochemistry

Liver and colon from animals subjected to TTR siRNA treatment and respective controls were excised, post-fixed in 10% formalin, embedded in paraffin and cut longitudinally at 3 μm. Colon from WT and KO mice was used as controls (*n* = 4). Histoclear (National Diagnostics, Atlanta, GA, USA) was used to deparaffinate sections that were thereafter hydrated in a descent alcohol series. Endogenous peroxidase activity was inhibited with 3% hydrogen peroxide in methanol and sections were blocked with 10% fetal bovine serum and 0.5% Triton x-100, in phosphate buffer saline. Primary antibodies against human TTR (1:600, rabbit polyclonal, DAKO, Glostrup, Denmark) and mouse Ttr (1:1500, rabbit polyclonal, Q-Biogen llkirch Cedex, France) were used. After incubation with secondary antibody (anti-rabbit IgG, 1:200, Vector, Burlingame, CA, USA), slides were incubated with avidin-biotin-peroxidase complex (ABC Elite, Vector) and visualized using 3.3′-diaminobenzidine as a chromogen.

### Immunofluorescent double labeling

For double immunofluorescence analyses, sciatic nerve, DRG and colon from Hsf/V30M animals were excised and processed as described above. Human sural nerve biopsies were also used. Rabbit polyclonal anti-human TTR (1:100, DAKO), sheep polyclonal anti-human TTR (1:100, Abcam, Cambridge, UK), mouse monoclonal anti-heparan sulfate proteoglycans (1:100, Amsbio, Tokyo, Japan), goat polyclonal anti-Octamer transcription factor 6 (Oct-6, 1:25, Santa Cruz Biotechnology, Santa Cruz, CA, USA), rabbit polyclonal anti-S100 (1:100, DAKO), rabbit polyclonal anti-early endosome antigen 1 (EEA1, 1:100, Sigma-Aldrich, St. Louis, MO, USA) and mouse monoclonal anti-lysosomal-associated membrane protein 1 (Lamp1, 1:75, Abcam) were used as primary antibodies. Secondary antibodies included donkey anti-rabbit Alexa Fluor 488, donkey anti-goat Alexa Fluor 568, donkey anti-sheep Alexa Fluor 488, goat anti-rabbit Alexa Fluor 568, goat anti-mouse Alexa Fluor 568 and 488 (1:1,000 Molecular Probes, Oregan, USA). Slides were mounted with Vectashield containing 4’.6-diamino-2-phenylindole (DAPI) (Vector) and visualized in a laser scanning Confocal Microscope Leica TCS SP5 II (Leica Microsystems, Heidelberg, Germany).

TTR-immunopositive SC in human sural nerve biopsies were detected by merged images with anti-TTR antibody and a SC marker (Oct-6). The number of TTR-immunopositive SC in each group was calculated as an average of 5 visual fields (447.63 μm × 335.40 μm) per sample (*n* = 4 per group) at an original magnification of 20x, in a Axio Imager (Zeiss, Hertfordshire, UK).

### Statistical analysis

Two or three groups’ comparison was performed with Student T-test or One-way ANOVA, respectively. For One-way ANOVA, Bonferroni was used as the post test. Data are expressed as mean values ± standard error of the mean (SEM) and *p*-values of less than 0.05 were considered to be significant (p* < 0.05, p** < 0.01 and ***p < 0.001).

## Results

### Non-fibrillar TTR is produced and degraded by Schwann cells in the FAP mouse model Hsf/V30M

Expression of *TTR* by SC of sciatic nerve in a mouse model carrying the TTR V30M mutation but missing TTR deposition in the PNS and ANS was previously described [25]. Thus, we questioned whether this feature is recapitulated in an FAP mouse model deficient for the *Hsf-1* (Hsf/V30M). In this model, non-fibrillar TTR deposition is widespread along the gastrointestinal tract since the first month of age. Furthermore regarding the nervous system, TTR deposits are found in the extracellular matrix of sciatic nerve, DRG and parasympathetic ganglia approximately at 6 months of age [22]. For this reason, animals with 6 months-old were chosen for subsequent analyses.

**Figure 1:**
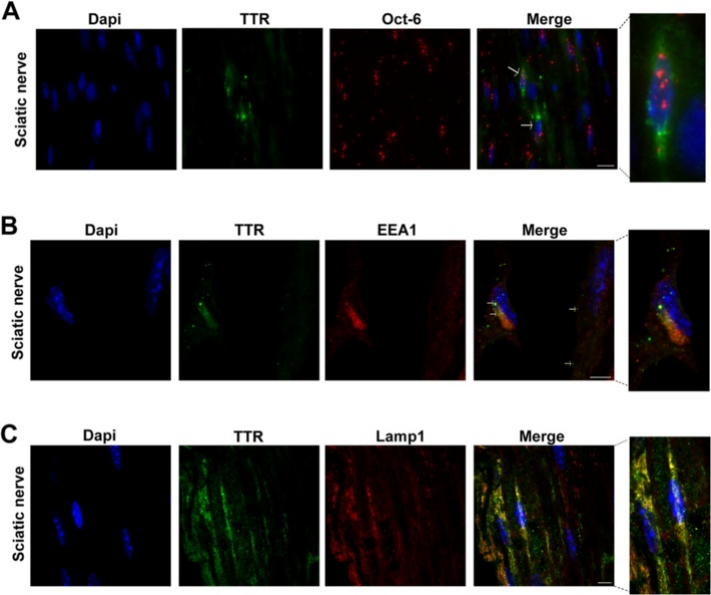
**SC express and internalize TTR in the Hsf/V30M FAP mouse model. A)** Representative picture of double immunofluorescence between TTR (green) and Oct-6 (red) in Hsf/V30M nerves, demonstrating TTR intracellular staining in SC (arrows). Superposition of the labels, with DAPI (blue), is shown (merge, *n* = 4). Scale bar 10 μm. **B)** Double immunofluorescence of TTR (green) and EEA1 (red) demonstrating colocalization between the 2 proteins in SC of Hsf/V30M mice (arrows). Scale bar 5 μm. **C)** Confocal representative image of a sciatic nerve section stained with anti-TTR antibody (green) and a lysosome marker (Lamp1; red). Colocalization between both markers was noticed in yellow; scale bar 5 μm.

Using qPCR, *TTR* expression by the sciatic nerve of Hsf/V30M mice was found (61.7 units ratio of *TTR* mRNA and *Gapdh* mRNA ± 16.2 SEM). With double immunofluorescence between TTR and Oct-6, a SC marker, TTR intracellular staining in these cells was noticed (Figure 1A, arrows), suggesting them as the primary source of *TTR* in nerve, in accordance with previous results from other FAP mouse models [25,26].

To localize TTR within SC, additional studies were performed using double immunofluorescence between TTR and a marker for early endosome antigen 1 (EEA1). Looking for cells previously stained with Oct-6, consecutive cross-sectional images revealed that indeed SC presented intracellular punctuate material, visible in higher magnification, that colocalize with EEA1 (Figure 1B, arrows). Further labeling with TTR and Lamp1 demonstrated colocalization of V30M TTR with lysosomes (Figure 1C), indicating that SC, might also be important for TTR clearance.

### Non-fibrillar TTR is internalized by glial cells of sensory ganglia

**Figure 2:**
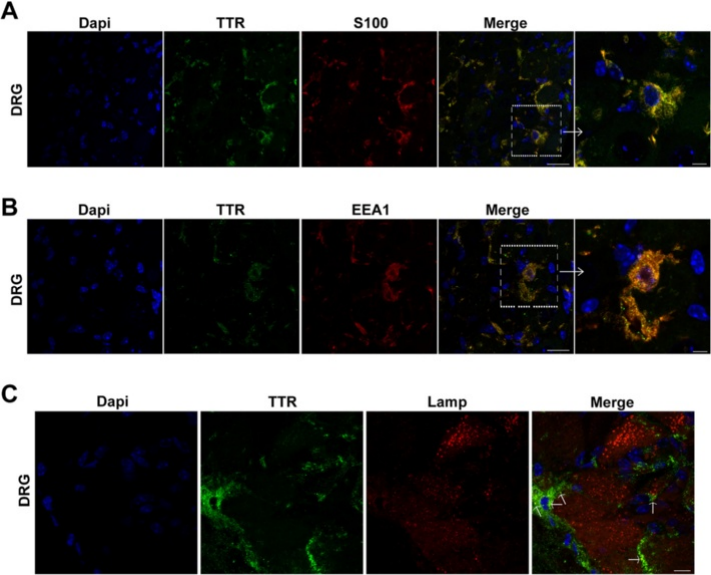
**Glial cells from DRG internalize TTR, in a mouse model of FAP. A)** Double immunofluorescence of TTR (green) and S100 (red), a marker for glial cells, in DRG of Hsf/V30M mice, showing TTR localized inside satellite cells. Scale bar 20 μm; higher magnification 5 μm. **B)** TTR colocalizing with EEA1 in satellite cells from DRG of Hsf/V30M animals. Scale bar 20 μm; higher magnification 5 μm. **C)** Representative confocal image demonstrating colocalization between TTR (green) and lysosomes (Lamp1; red) in satellite cells from DRG (arrows). Scale bar 8 μm.

Data reported so far indicate that SC, besides being a source of *TTR* might also be important for TTR clearance in the sciatic nerve. We next investigated TTR immunoreactive glial cells from DRG of Hsf/V30M mice. In fact, with double immunofluorescence between TTR and S100, TTR intracellular staining was found in glial cells of DRG (possible satellite cells; Figure 2A). Since it was previously shown that *TTR* is not synthesized by cells of DRG [27] and in this work we found colocalization of TTR with EEA1 (Figure 2B), we hypothesized that satellite cells have an active role in TTR internalization. Moreover, we found TTR signal partially colocalized with lysosomes (Figure 2C); therefore we can conclude that these cells participate in TTR clearance in this animal model.

### Intracellular TTR in human Schwann cells

**Figure 3:**
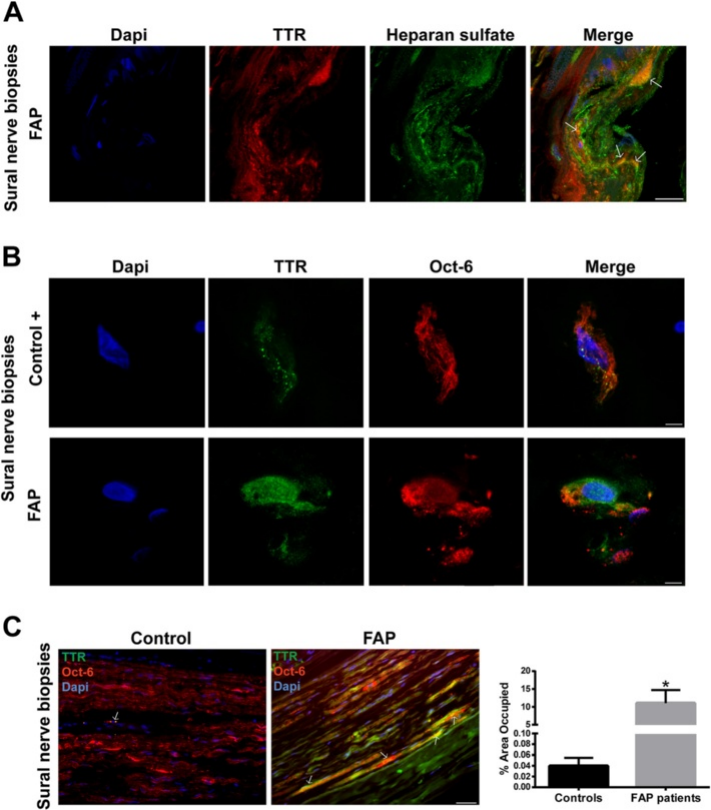
**Intracellular TTR in SC of human nerve biopsies. A)** Representative photomicrographs obtained with confocal microscopy showing colocalization (yellow and orange in merged panel) between TTR (red) and heparan sulfate (green) in sural nerve biopsies from FAP patients. Scale bar 15 μm. **B)** Representative images from double immunofluorescence between TTR (green) and Oct-6 (red) in sural nerve biopsies from disease control individuals and FAP patients, demonstrating TTR intracellular staining in SC. Superposition of the labels, with DAPI (blue), is shown (merge, *n* = 4). Scale bar 4 μm. **C)** Low magnification images from longitudinal sections of human sural nerves showing colocalization between TTR and Oct-6 (arrows); scale bar 40 μm. Chart represents quantification of TTR positive SC, related to total SC in nerve sections. Results are presented as means ± SEM (p* < 0.05).

**Figure 4:**
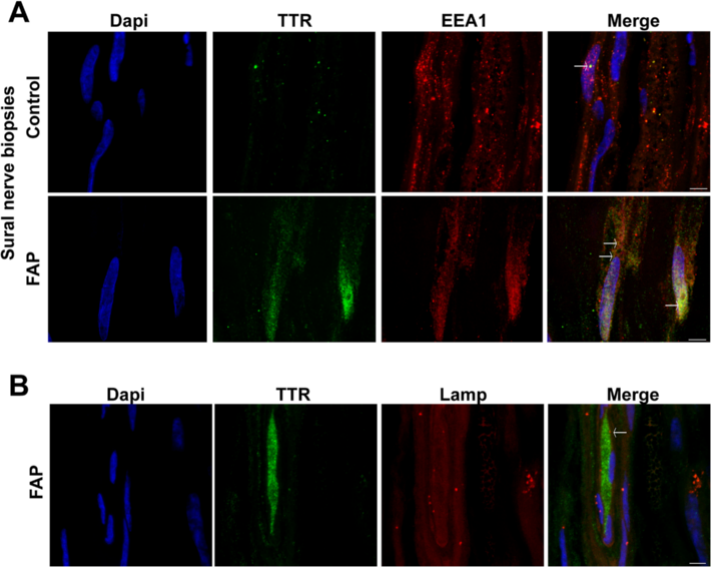
**TTR is internalized by human SC. A)** Double immunofluorescence of TTR (green) and EEA1 (red) showing colocalization spots between the 2 proteins in SC of FAP patients and normal control individuals (arrows). Scale bar 4 μm. **B)** Confocal representative image showing scarce colocalization between TTR (green) and lysosomes (red). Scale bar 3 μm.

It was previously found that TTR fibril extracts contain heparan sulfate proteoglycans [28,29]. Thus, we started by characterizing TTR deposits on sural nerve biopsies from FAP patients regarding this glycosaminoglycan. As illustrated in merged panel of Figure 3A, heparan sulfate colocalized with TTR deposits, demonstrating that heparan sulfate is present in TTR deposits as co-localized molecules. Following the previous results with the FAP mouse model, we wondered if intracellular TTR could also be observed in nerves from FAP patients. To address this issue, we also used sural nerve biopsies from normal individuals (disease free) as controls. In disease control individuals, the majority of SC were negative for WT TTR intracellular staining; surprisingly, few positive cells presented WT TTR immunoreactive punctuate material (Figure 3B, top panel, and 3C), suggesting that SC might have an important role for uptake and degradation of WT TTR reaching the nerve though the blood stream. In FAP carriers, some SC had generalized V30M TTR intracellular staining (Figure 3B, bottom panel, and 3C) and the percentage of TTR positive SC was significantly higher than for disease control patients (Figure 3C). TTR colocalization with EEA1 was observed in both cases (Figure 4A, arrows), corroborating the notion that punctuate intracellular material arise from TTR internalization and not from cell synthesis. Colocalization between TTR and lysosomes was scarce (Figure 4B, arrow), most likely related to sample storage and processing

.

### Intracellular TTR in satellite cells from the myenteric plexus

**Figure 5:**
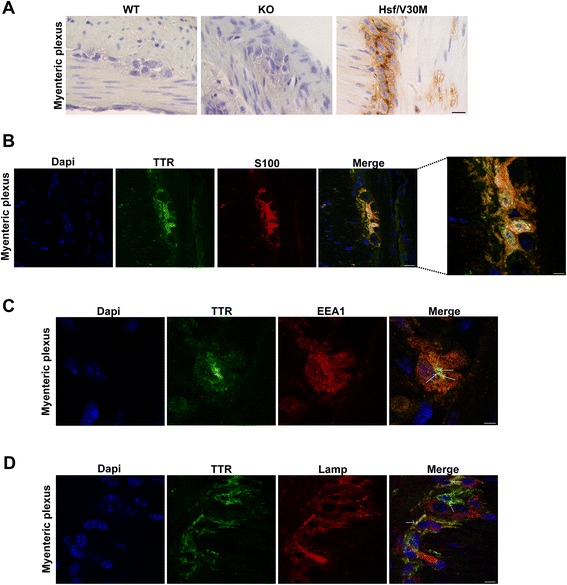
**TTR is internalized by satellite cells from the myenteric plexus. A)** TTR immunohistochemical staining on colon myenteric plexus from WT, TTR KO and Hsf/V30M animals. Scale bar 20 μm. **B)** Double immunofluorescence between TTR (green) and S100 (red), a satellite cell marker, showing TTR inside these cells. Scale bar 14 μm; higher magnification 5 μm. **C)** Representative confocal image of myenteric plexus section showing a satellite cell with intracellular TTR staining (green) colocalizing with EEA1 (red) (arrows). Scale bar 4 μm. **D)** Confocal microscopy image with anti-TTR antibody (green) and lysosome marker (Lamp1; red). Nuclei stained blue with DAPI. Arrows indicate colocalization of TTR with lysosomes. Scale bar 5 μm.

**Figure 6:**
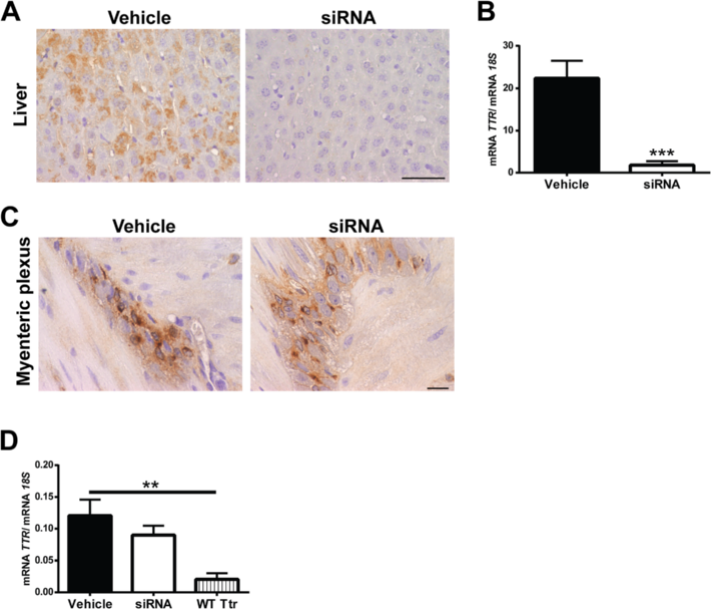
***TTR*** **is expressed by colon of the Hsf/V30M FAP mouse model. A)** Photomicrographs illustrating TTR staining in liver of Hsf/V30M mice, treated with TTR siRNA or vehicle; scale bar 50 μm. **B)** Histogram represents *TTR* mRNA levels in liver of Hsf/V30M mice treated with TTR siRNA as compared with control animals receiving only vehicle. Data was normalized using *18S* as the housekeeping gene (p*** < 0.001). **C)** Histological images of colonic myenteric plexus from Hsf/V30M mice treated with TTR siRNA as compared with vehicle treated animals. Scale bar 20 μm. **D)** Histogram denoting *TTR* mRNA expression in colon from Hsf/V30M mice treated with vehicle or siRNA and *Ttr* mRNA from WT animals. Data was normalized using *18S* as the housekeeping gene (p** < 0.01).

In FAP disease, ANS is severely affected by TTR deposition ultimately leading to bladder dysfunction, diarrhea, malabsorption and motility disturbances [14]. Thus, we questioned whether satellite cells from the myenteric plexus may also be able to internalize TTR in FAP. By histological analysis of Hsf/V30M mice colon, non-fibrillar TTR immunostaining was found in Auerbach plexus (myenteric plexus) especially inside smaller cells surrounding the nerve cell bodies (compatible with satellite cells), in contrast with WT and TTR KO animals that were negative for TTR (Figure 5A). Following these results, we performed double immunofluorescence between TTR and S100 in colon sections of Hsf/V30M animals. Colocalization between the two proteins was observed (Figure 5B). Since the TTR staining present in these cells partially colocalized with EEA1 (Figure 5C, arrows) and Lamp1 (Figure 5D, arrows), it is reasonable to suggest that satellite cells have an important role in non-fibrillar TTR uptake and internalization. However, we could not discard the possibility of *TTR* synthesis by these cells of myenteric plexus, especially when *TTR* production in colon was already previously suggested [30]. To address this question we treated 5 months-old Hsf/V30M mice with TTR siRNA, to inhibit TTR deposition in the gastrointestinal tract. With this treatment we were able to silence 92% *TTR* expression by the liver (Figure 6A and B), avoiding TTR circulation in plasma and burden in tissues, as previously described [31]. Nevertheless, TTR immunostaining in satellite cells from myenteric plexus was equally observed in animals treated with TTR siRNA or controls (vehicle treated mice) (Figure 6C). By qPCR we found *TTR* expression in colon of Hsf/V30M mice, with similar levels of relative expression between siRNA and vehicle treated mice (Figure 6D). Additionally, *Ttr* expression in colon from WT mice was considerably lower than in Hsf/V30M mice (Figure 6D). This, together with the fact that no TTR reactivity was found in satellite cells from myenteric plexus in WT mice, suggests that FAP colonic myenteric plexus might be able to synthesize and clear TTR.

## Discussion

FAP is a peculiar form of neuropathy with clinical symptoms generally occurring in the second or third decade of life. However, a late-onset form of disease has also been described, affecting individuals over 50 years of age and characterized by low penetrance rate and different pathological presentations [32]. Sensory impairment, wasting and weakness results from neuronal and axonal loss consequent to obliteration/dysfunction of small vessels supplying the nerve tissue, nerve fiber compression by TTR deposits or toxic effects of amyloid precursors [32,33]. Additionally, motor sensory type is often found in FAP, characterized by muscle atrophy with distal predominance and decreased motor conduction velocity [34].

In peripheral nerve, TTR deposits are predominantly found in the endoneurium, in close contact with SC and collagen fibrils [2]. Thus, an impairment of axon-SC interaction due to SC dysfunction might contribute to axonopathy. Similarly, in nervous ganglia, TTR deposition occurs in the stroma in close association to satellite cells [35-37]. Satellite cells covering sensory neurons are similar to the SC of peripheral nerve as they are both derived from the neural crest of the embryo during development [38]. They are known as glial supporting cells of the nervous system and have important roles on mechanical support to neurons, nutrients/oxygen supply and removal of cell debris [39]. *TTR* expression by glial cells of sensory neurons was previously suggested [40]. However, this hypothesis was later disputed and the results were attributed to TTR contamination by the meninges [27]. Recently, *TTR* gene expression by SC of peripheral nerve was reported [25]. Accordingly, in the present study, we also observed *TTR* expression in the peripheral nerve of Hsf/V30M mice, corroborating the previous data.

Glial cells regulate neurons microenvironment and appear to be actively engaged in the control of extracellular matrix composition [39]. Thus, several studies indicate the ability of these cells in the uptake of different substances [41-45]. Since TTR non-fibrillar deposits are found in the extracellular matrix in close association with glial cells, we decided to investigate whether these cells in sensory neurons and peripheral nerve might be important for TTR uptake *in vivo*. In fact we found immunoreactive TTR in satellite and SC, both in the Hsf/V30M transgenic model and in tissues from FAP patients. Occasionally, the observed intracellular TTR presented a punctate-like pattern, compatible with its presence within vesicles. Once in the cell, endocytic vesicles are rapidly targeted to the early endosomes. Thus, despite the existence of numerous internalization routes, early endosomes are a focal point of the endocytic pathway [46,47]. EEA1 is a widely used marker for early endosomes due to its colocalization with the transferrin receptor [48]. In this work, TTR colocalization with EEA1 and a lysosome marker (Lamp1) in glial cells of peripheral nerve and sensory ganglia indicate internalization and consequent vesicular transport of TTR through early endosomes for degradation.

In Alzheimer's disease, A*β* internalization by glia has been observed *in vitro* and *in vivo* [43,49] suggesting an important role for these cells in Alzheimer pathology. Nevertheless, exposure of glial cells to A*β* aggregates could have detrimental consequences with upregulation of inflammatory mediators and nitric oxide, resulting in glia and neuron cell death [50].

Another striking feature of FAP is severe autonomic nervous dysfunction, affecting particularly the gastrointestinal tract [14,32]. Initial gastrointestinal symptoms are severe constipation alternating with periods of diarrhea, nausea and vomiting [51]. Evidence suggests that environmental and genetic factors have impact on the clinical pattern of FAP [52]. For instance, in Japanese FAP patients no significant destruction of the enteric nervous system is observed [53] while Portuguese FAP patients present infiltration of amyloid material in the space between two adjacent ganglia, accompanied by different degrees of neuronal loss [54]. However, the mechanisms leading to neuron cell death remain poorly understood. Some factors that have been associated with the pathogenesis of gastrointestinal dysfunction are a depletion of neuroendocrine cells, such as serotonin, somatostatin or PYY immunoreactive cells [55], accumulation of advanced glycation end products [56], loss of interstitial cells of Cajal [57] and amyloid deposition in sympathetic ganglia [58]. Although amyloid deposits have not been found in the myenteric plexus of Hsf/V30M FAP mouse model, this was the first animal model presenting non-fibrillar TTR deposition in the autonomic nervous system of the GI tract [22]. Therefore, we next investigated whether satellite cells were able to internalize TTR, also in this system. TTR colocalization with EEA1 and lysosomes indicated that endocytic trafficking pathways are activated in satellite cells from the myenteric plexus of Hsf/V30M mice. Importantly, this is the first study showing *TTR* synthesis by the enteric tissue, in this FAP mouse model. Therefore, it is reasonable to suggest that satellite cells may also be synthesizing mutated *TTR* which in turn might contribute for the non-fibrillar TTR deposition. It would be interesting to confirm these results in biopsy colon specimens from FAP patients, however such specimens are very difficult to obtain since the conditions of these patients not often calls for colonoscopy.

Overall, the present study brings to light new insights into the FAP pathophysiology. Besides TTR endocytosis by fibroblasts demonstrated both *in vitro* and *in vivo* [20], TTR internalization *in vivo* by glial cells of peripheral nerve, sensory ganglia and myenteric plexus was here demonstrated. However, whether TTR uptake by these cells is neuroprotective or neurotoxic leading to glial and neuronal cell death with autonomic dysfunction needs further investigation. Furthermore, additional studies are needed to clarify the molecular mechanisms and signaling platforms involved in these particular systems.

## Conclusions

The results presented in the current study confirm previous findings that *TTR* is expressed on peripheral nerve and colon, possibly by glial cells. SC and satellite cells from sciatic nerve, DRG and myenteric plexus are able to internalize and degrade TTR in the Hsf/V30M mouse model, contributing for TTR extracellular clearance. Additionally, TTR colocalization with EEA1 was found in SC from human patients, suggesting a dual role for these cells in FAP. Therefore, an imbalance of this system might trigger or accelerate TTR aggregates deposition in target tissues. These novel data regarding the physiopathology of FAP might open new windows of action in the design of new therapeutic targets.

## Abbreviations

ANS: Autonomic nervous system
DRG: Dorsal root ganglia
EEA1: Early endosome antigen-1
FAP: Familial amyloidotic polyneuropathy
Gapdh: Glyceraldehyde 3-phosphate dehydrogenase
Hsf-1: Heat shock factor 1
KO: Knockout
Lamp1: Lysosomal-associated membrane protein 1
PNS: Peripheral nervous system
qPCR: Real-time polymerase chain reaction
SC: Schwann cells
SEM: Standard error of the mean
TTR: Transthyretin
WT: Wild-type
RNAi: Interference RNA
